# The Profile of T Cell Responses in Bacille Calmette–Guérin-Primed Mice Boosted by a Novel Sendai Virus Vectored Anti-Tuberculosis Vaccine

**DOI:** 10.3389/fimmu.2018.01796

**Published:** 2018-08-03

**Authors:** Zhidong Hu, Ling Gu, Chun-Ling Li, Tsugumine Shu, Douglas B. Lowrie, Xiao-Yong Fan

**Affiliations:** ^1^Shanghai Public Health Clinical Center, Key Laboratory of Medical Molecular Virology of MOE/MOH, Fudan University, Shanghai, China; ^2^School of Laboratory Medicine and Life Science, Wenzhou Medical University, Wenzhou, China; ^3^ID Pharma, Ibaraki, Japan

**Keywords:** tuberculosis, Sendai virus, vaccine, T cell responses, prime-boost, IL-2

## Abstract

The kinds of vaccine-induced T cell responses that are beneficial for protection against *Mycobacterium tuberculosis* (*Mtb*) infection are not adequately defined. We had shown that a novel Sendai virus vectored vaccine, SeV85AB, was able to enhance immune protection induced by bacille Calmette–Guérin (BCG) in a prime-boost model. However, the profile of T cell responses boosted by SeV85AB was not determined. Herein, we show that the antigen-specific CD4^+^ and CD8^+^ T cell responses were both enhanced by the SeV85AB boost after BCG. Different profiles of antigen-specific po T cell subsets were induced in the local (lung) and systemic (spleen) sites. In the spleen, the CD4^+^ T cell responses that were enhanced by the SeV85AB boost were predominately IL-2 responses, whereas in the lung the greater increases were in IFN-γ- and TNF-α-producing CD4^+^ T cells; in CD8^+^ T cells, although IFN-γ was enhanced in both the spleen and lung, only IL-2^+^TNF-α^+^CD8^+^ T subset was boosted in the latter. After a challenge *Mtb* infection, there were significantly higher levels of recall IL-2 responses in T cells. In contrast, IFN-γ-producing cells were barely boosted by SeV85AB. After *Mtb* challenge a central memory phenotype of responding CD4^+^ T cells was a prominent feature in SeV85AB-boosted mice. Thus, our data strongly suggest that the enhanced immune protection induced by SeV85AB boosting was associated with establishment of an increased capacity to recall antigen-specific IL-2-mediated T cell responses and confirms this Sendai virus vector system as a promising candidate to be used in a heterologous prime-boost immunization regimen against TB.

## Introduction

Tuberculosis (TB) remains among the most deadly health challenge to humankind. Bacille Calmette–Guérin (BCG), the attenuated form of *Mycobacterium bovis*, has been used for over 80 years to protect children against severe forms of TB (1). However, its protective efficacy against pulmonary TB was found to vary from 0 to 80% in adults (2), hence a more effective vaccine is needed.

T cell responses are regarded as a critical factor in containment of *Mycobacterium tuberculosis* (*Mtb*) infection. After phagocytosis, *Mtb* preferentially resides in phagosomes, where its antigens are processed and assembled onto MHC-II molecules for presentation to CD4^+^ T cells (3). During the course of *Mtb*-driven differentiation, the T cells can gain the capacity to simultaneously produce two or more key cytokines and are called poly-functional T cells, which are considered to be superior effectors of protective immunity as compared to cells that produce only one cytokine (4). Specially, IFN-γ, IL-2, and TNF-α secreting CD4^+^ T cells, which are known as Th1 cells, are regarded as crucial for activation of effector functions to control intracellular *Mtb* and are correlated with protection (5, 6).

Although the role of CD8^+^ T cell-mediated immune responses against TB infection is less well defined than that of Th1 CD4^+^ T cells, these cells are also considered to play a crucial role in optimal immunity and protection. It was shown that CD8^+^ T cells were essential against *Mtb* infection in the models of mice (7, 8), cattle (9), and macaques (10). Furthermore, vaccine-induced antigen-specific CD8^+^ T cell responses were found to contribute to strong or modest immune protection in several studies (11–14). Recently, we reported that a novel Sendai virus vectored vaccine, SeV85AB, induced robust T cell responses and substantial protection against *Mtb* infection, which was mainly mediated by CD8^+^ T cells (15).

Insufficient induction of T cell responses by BCG immunization might underlie the vaccine’s inadequacies and boosting these responses by novel vaccines might be an appropriate vaccine strategy (16, 17). However, which kind of T cell responses are beneficial for the anti-TB immune protection remains controversial (18, 19); notably, the classical marker, IFN-γ, was found to play a minor role in, or even be detrimental to, the anti-TB immunity (20–22). Although we had shown that intra-nasal delivery of the SeV85AB vaccine was able to enhance immune protection induced by BCG in a prime-boost model (15), the profile of T cell responses boosted by SeV85AB was not determined. Herein, we show that SeV85AB boosting established substantial T cell responses in the lung that differed from systemic immunity; there were different profiles of antigen-specific poly-functional T cell subsets in the lung compared with the spleen. After challenge by *Mtb* infection, SeV85AB-boosted mice had significantly higher levels of recall CD4^+^ and CD8^+^ T cell responses, which were mainly mediated by IL-2. In contrast, the IFN-γ-producing cells were barely boosted by SeV85AB. The proportion of cells with central memory phenotype of peptides-responding CD4^+^ T cells was elevated in SeV85AB-boosted mice after *Mtb* challenge. Our study, therefore, lends strong support to the adoption of Sendai virus as a promising vector system to be used in a heterologous prime-boost immunization regimen against TB.

## Materials and Methods

### Animals and Immunization

This study was approved by the Institutional Animal Care and Use Committee and was performed according to the guidelines of the Laboratory Animal Ethical Board of Shanghai Public Health Clinical Center. Specific pathogen-free female BALB/c mice aged 6–8 weeks were immunized with BCG subcutaneously [s.c., 5 × 10^6^ CFU (colony forming units), in 100 µl PBS] in each hind leg and boosted intra-nasally (i.n.) with SeV85AB [1 × 10^7^ cell infectious units (CIU), in 20 µl PBS]. BCG, SeV85AB single immunizations, and PBS were used as controls. For the evaluation of primary cellular immune responses, 4 weeks after vaccination, animals were sacrificed, then, the lungs and spleens were aseptically removed for antigen-specific T cell immune response assessments. For the evaluation of recall immune responses after infection, the mice were challenged through a respiratory route by the *Mtb* virulent strain H37Rv 4 weeks after immunization and maintained in a level 3 bio-containment animal facility. Five weeks later, the mice were sacrificed and lungs sampled to assess recall responses by intracellular staining (ICS) as described below.

### Harvest of Splenocytes and Lung Cells

Spleen was mechanically disrupted and single splenocytes were filtered, and then subjected to red blood cell lysis. Lung was gently minced by scissors and then incubated with DNase I (10 U, Thermo) and collagenase IV (1 mg/ml, Invitrogen) in 10 ml R10 medium (RPMI-1640 medium containing 10% fetal bovine serum and 1% Penicillin and Streptomycin) for 30 min at 37°C. The collagenase-digested tissue pieces were filtered through a 70 µm cell strainer (Fisher Scientific) by gently squashing with the plunger of a syringe. The cell suspension was centrifuged and red blood cells were lysed. The single splenocytes and lung lymphocytes were washed and re-suspended in R10 medium.

### IFN-γ Enzyme Linked Immunospot (ELISPOT) Assay

Enzyme-linked immunospot assays were performed according to IFN-γ ELISPOT kit protocols (BD Biosciences). Briefly, 96-well plates were coated with anti-mouse IFN-γ antibody at 4°C overnight. Then, they were washed and blocked with R10 medium at room temperature for 2 h. Isolated lung cells or splenocytes were added at 2 × 10^5^ cells per well with peptide pools (5 µg/ml) or PPD (10 µg/ml). PMA (50 ng/ml, Sigma) and ionomycin (1 µg/ml, Sigma) stimulation were used as positive controls. The cells were stimulated at 37°C for 20 h. The cell suspension was aspirated and washed with PBST (PBS containing 0.5% Tween-20), and then incubated with anti-mouse IFN-γ biotinylated detection antibodies for 2 h. The cell suspension was aspirated and washed with PBST and then incubated with streptavidin-horseradish superoxidase conjugated anti-biotin antibodies for an additional 1 h. After washing with PBST and PBS, AEC substrate solution was added and incubated for 30 min before rinsing away with water. The plates were air-dried and analyzed with Immunospot Reader (Champspot III, Beijing Sage Creation Science).

### Peptides

The peptides were synthesized by GL Biochem (Shanghai, China) with 95% purity. MPVGGQSSF, 70-78aa; TFLTSELPGWLQANRHVKPT, 99-118aa; GLSMAASSALTL, 124-125aa and YAGAMSGL, 145-152aa were used as an Ag85A peptide pool. IYAGSLSAL, 144-152aa; ALLDPSQGMGPSLIG, 151-165aa; GPSSDPAWERNDPTQQIP, 181-198aa and HSWEYWGAQLNAMKGDLQ, 262-279aa were used as an Ag85B peptide pool.

### Intracellular Staining (ICS) and Flow Cytometry Analysis

The splenocytes or lung cells were stimulated with Ag85AB peptide pool (5 µg/ml) or PPD (10 µg/ml) in 96-well plates at 37°C for 1 or 14 h, respectively. Then, cells were incubated for an additional 5 h after the addition of 1 µl/ml Monensin and Brefeldin A (BD Biosciences), PMA (50 ng/ml), and inonomycin (1 µg/ml) stimulation were used as positive controls. After stimulation, cells were washed with wash PBS containing 2% fetal bovine serum and then incubated on ice with the mixture of antibodies for 30 min, then washed and subjected to fixation and permeation with fix/perm buffer (BD Bioscience). After washing, the cells were incubated with antibodies against intracellular cytokines on ice for another 30 min. Then, the cells were re-suspended for flow cytometry analysis (LSRFortessa, BD Biosciences). At least 50,000 T cells were harvested.

### Antibodies

The following antibodies were used in this study: CD3-Pacific Blue (clone 17A2) from BioLegend, and CD44-FITC (clone IM7), CD62L-Percp-Cyanine 5.5 (clone MEL-14), TNF-α-PE-Cyanine 7 (clone MP6-XT22) from eBioscience, and CD4-Alexa Fluor 647 (clone RM4-5), IFN-γ-APC-Cyanine 7 (clone XMG1.2), IL-2-PE (clone JES6-5H4) from BD Biosciences.

### Statistical Analysis

The statistical analysis was performed using GraphPad Prism 6 software. One-way ANOVA was used to determine the statistical significance for comparison of multiple groups, and two-way ANOVA was used for the grouped analysis.

## Results

### Boosting With SeV85AB Increased the IFN-γ Responses Primed by BCG Vaccination

**Figure 1:**
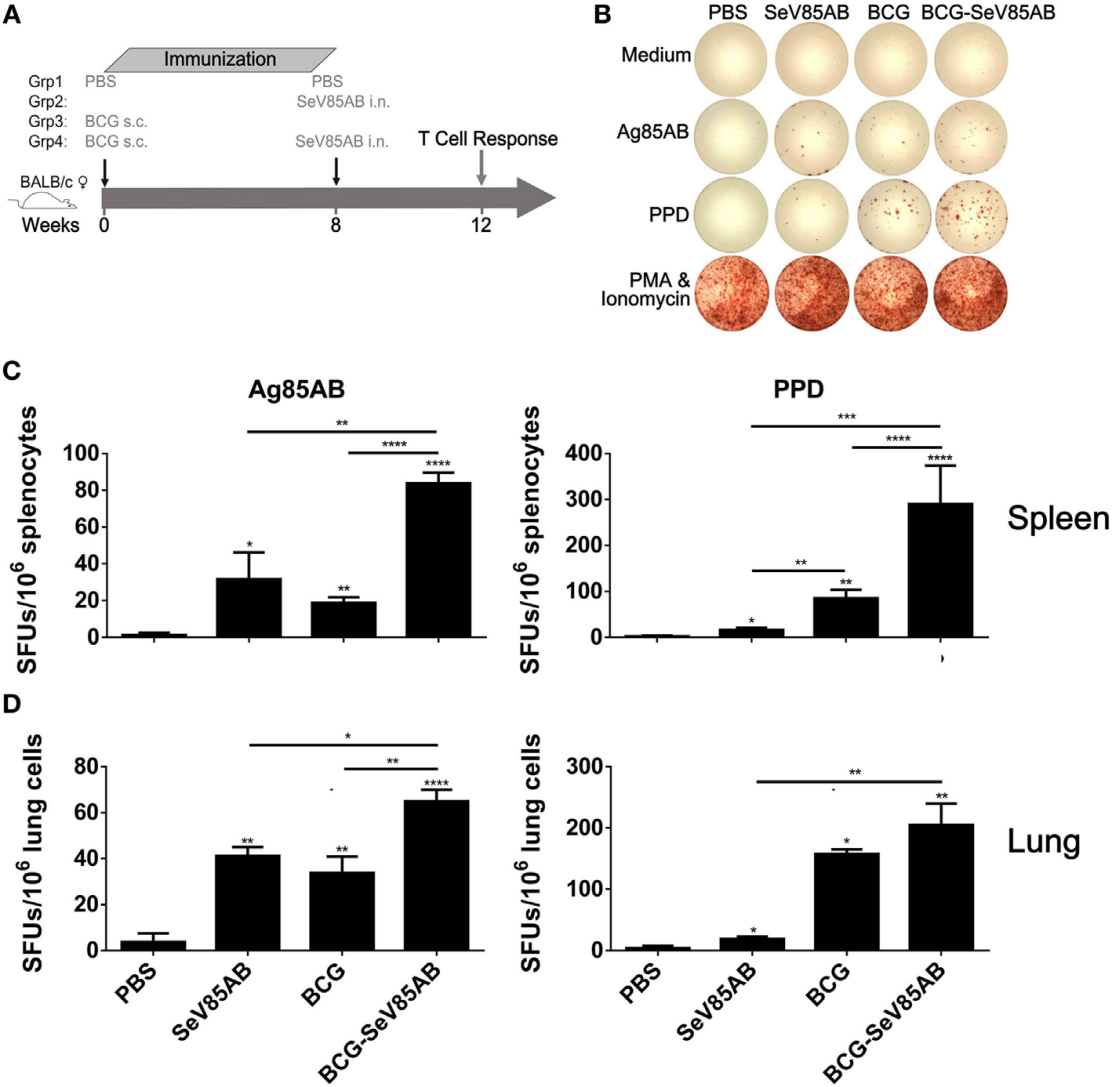
Boosting with SeV85AB increased the IFN-γ responses primed by bacille Calmette–Guérin (BCG) vaccination. **(A)** Immunization and detection schedule. Mice primed by BCG were i.n. boosted with SeV85AB or not, and 4 weeks later, they were sacrificed for assays of cellular immune responses. Controls received PBS, SeV85AB, or BCG only, at the indicated time points. **(B–D)** The IFN-γ responses determined by enzyme linked immunospot assay. Representative dot plots are shown in **(B)**. Cells from spleen **(C)** and lung **(D)** were stimulated for 20 h with Ag85AB peptide pool (5 µg/ml) or PPD (10 µg/ml) in 96-well plates and then the IFN-γ-secreting cells were detected and counted. PMA plus Ionomycin stimulation was used as positive control. Data are representative of two independent experiments with at least four mice per group. *P < 0.05, **P < 0.01, ***P < 0.001, and ****P < 0.0001.

The experiment protocol is shown diagrammatically in Figure 1A. Typical IFN-γ ELISPOT results are shown in Figure 1B. As expected, SeV85AB vaccination induced Ag85AB-specific immune responses, and this effect was significantly greater in BCG-primed mice, both in the spleen (Figure 1C) and in the lung (Figure 1D). Although PPD-specific cell responses were only slightly induced by SeV85AB, mice receiving SeV85AB boost secreted higher levels of IFN-γ compared with SeV85AB or BCG single immunization (Figures 1C,D).

### SeV85AB Boost Increased Systemic Poly-Functional T Cell Responses Primed by BCG Vaccination

**Figure 2:**
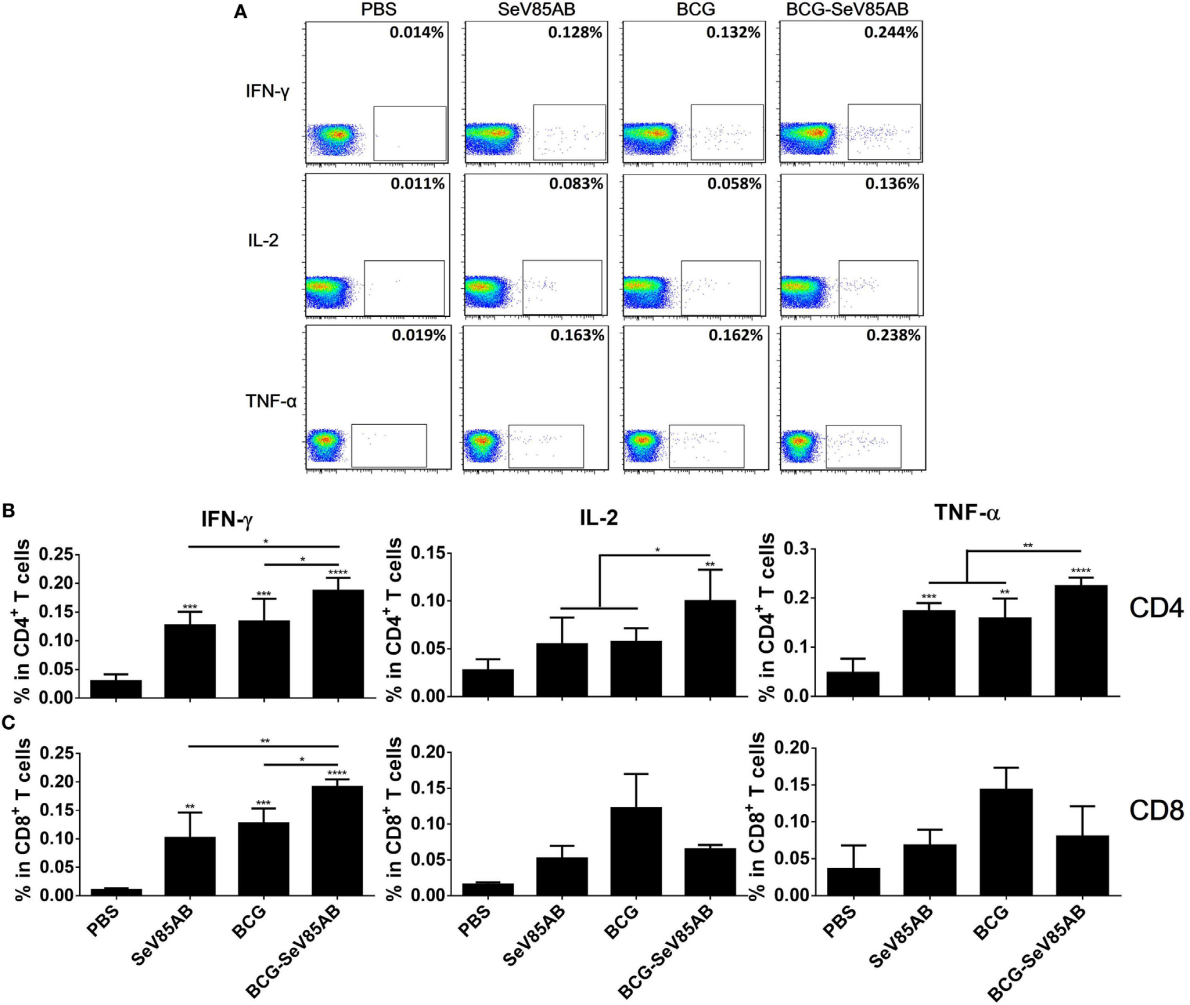
Flow cytometric analysis of ICS in the splenocytes of vaccinated mice. Splenocytes were collected 4 weeks after the last inoculation, incubated with Ag85AB peptides (5 µg/ml) in the presence of monensin and brefeldin A, and analyzed for cytokine production by ICS assay. CD3^+^CD4^+^ cells (CD4^+^ T cells) and CD3^+^CD4^−^ cells (CD8^+^ T cells) producing IFN-γ, IL-2, and TNF-α were analyzed. Representative flow cytometric plots of intracellular staining in CD4^+^ T cells are shown in **(A)**, and summary data of single cytokine producing CD4^+^ T cells **(B)** and CD8^+^ T cells **(C)** are shown with significant differences indicated. Data are representative of two independent experiments with at least four mice per group. *P < 0.05, **P < 0.01, ***P < 0.001, and ****P < 0.0001.

**Figure 3:**
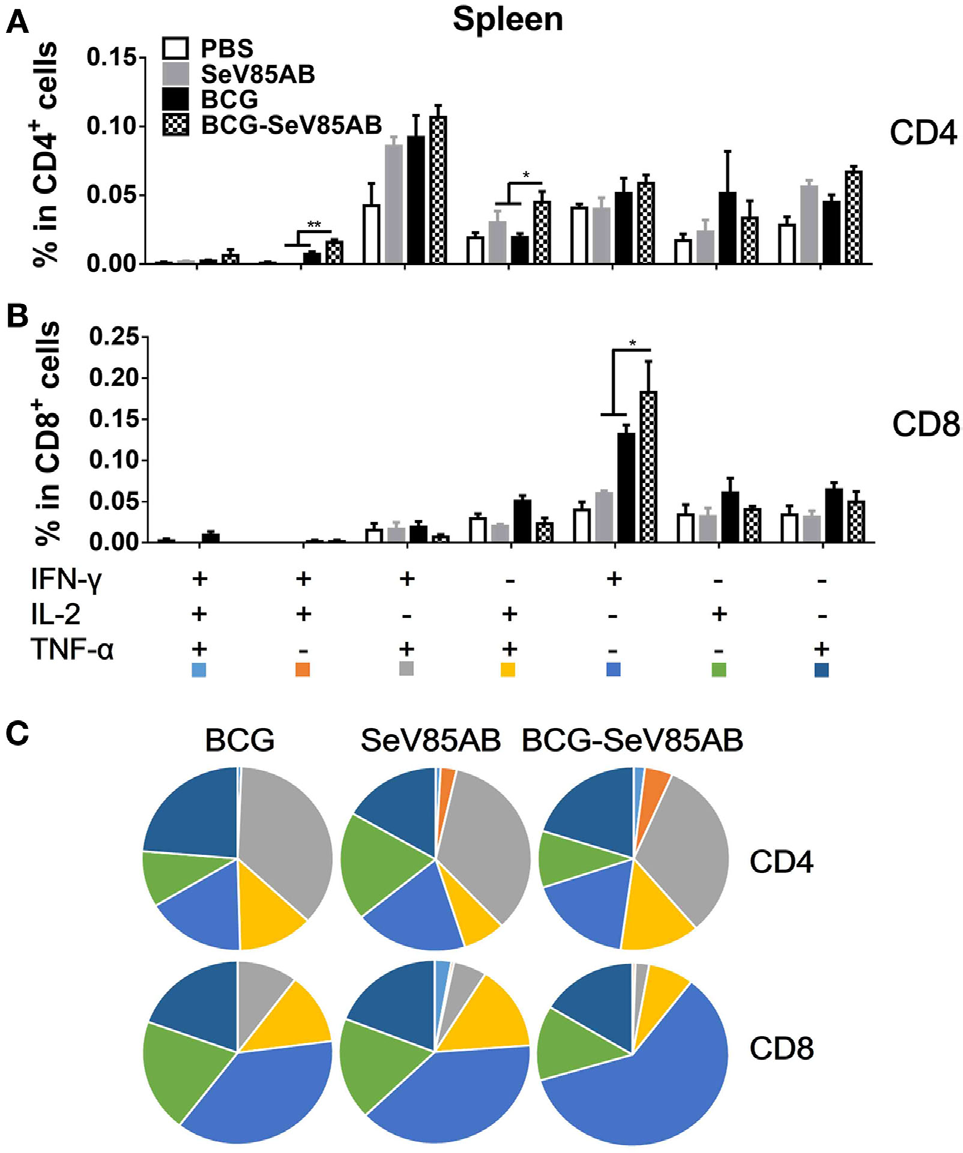
Characterization of poly-functional Ag85AB-specific T cell responses in the spleen. T cells producing IFN-γ, IL-2, and TNF-α were distinguished by ICS assay as seven subpopulations based on the production of these three cytokines in any combination. The percentage of subpopulations as components of the total CD4^+^ **(A)** or CD8^+^ **(B)** T cell population and the pie chart analysis **(C)** are shown. Significant differences in frequency of poly-functional T cell subsets are indicated. Data are representative of two independent experiments with at least four mice per group. *P < 0.05 and **P < 0.01.

At 4 weeks after vaccination, splenocytes were obtained from the different vaccination groups and stimulated with the Ag85AB peptide pool or incubated with medium as control (The gating strategy is shown in Figure S1 in Supplementary Material, CD4^+^ T cells were gated as CD3^+^CD4^+^ cells and CD8^+^ T cells were defined as CD3^+^CD4^−^ cells) and the results of flow cytometric ICS detection of IFN-γ, IL-2, and TNF-α positive T cells were compared between groups (representative mono-positive dot plots are shown in Figure 2A and dual-positive plots are shown in Figure S2 in Supplementary Material). Consistent with the ELISPOT assay, the production of all three cytokines in CD4^+^ T cells was significantly enhanced by SeV85AB boost (Figure 2B), whereas only IFN-γ was increased in CD8^+^ T cells (Figure 2C). The SeV85AB boost induced higher levels of antigen-specific poly-functional CD4^+^ T cell responses, in which the increased percentage of dual-positive IFN-γ^+^IL-2^+^ and IL-2^+^TNF-α^+^ cells were statistically significant (Figures 3A,C). Additionally, the mono-positive IFN-γ^+^CD8^+^ subset was significantly increased (Figures 3B,C).

### SeV85AB Boost Increased Poly-Functional T Cell Responses in the Lung Primed by BCG Vaccination

**Figure 4:**
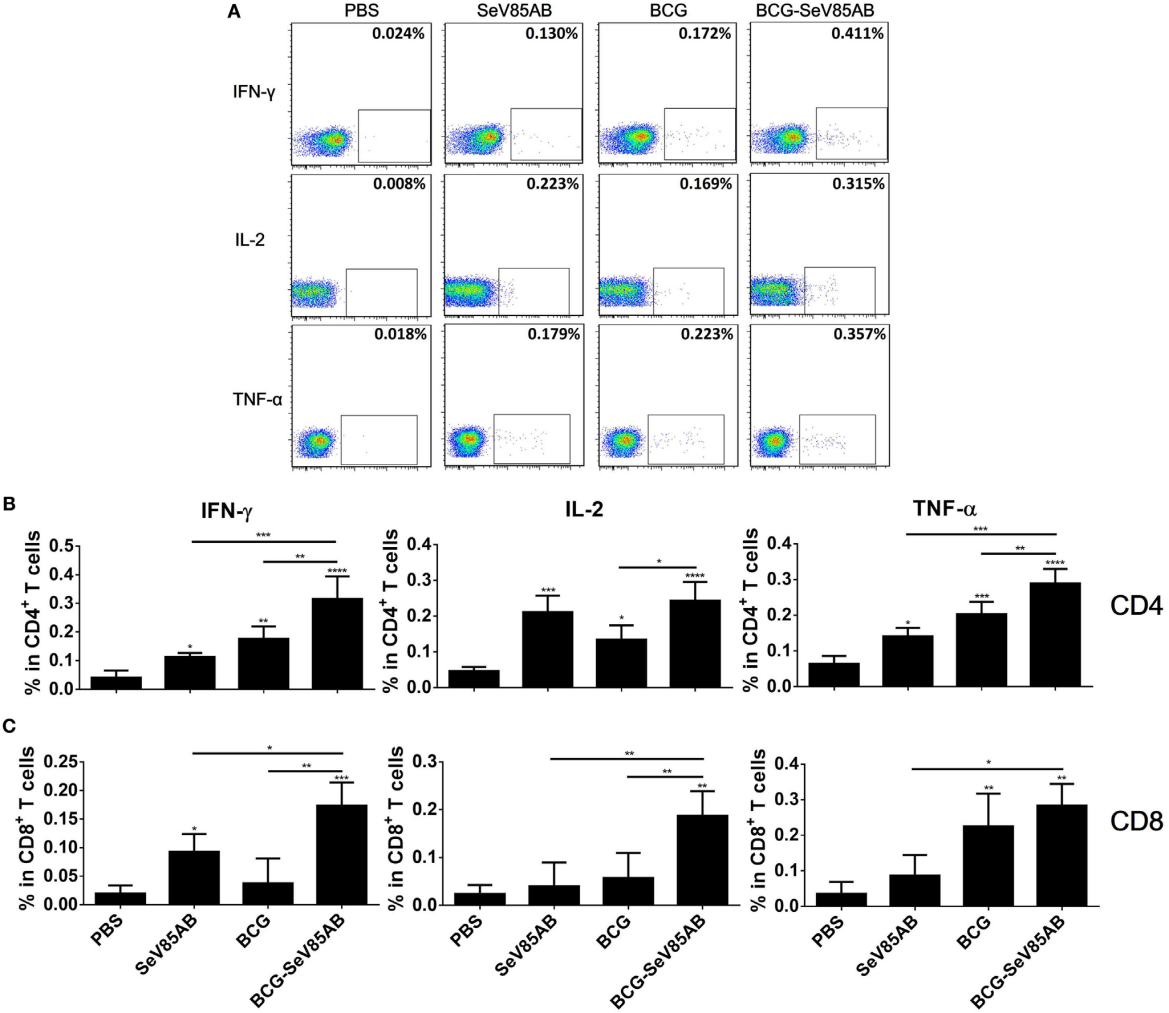
Flow cytometric analysis of ICS in the lung cells of vaccinated mice. Lung cells were collected, stimulated, and analyzed for cytokine production by ICS assay as described for splenocytes in Figure 2. Representative flow cytometric data plots from CD4^+^ T cells **(A)** and summarized data of single cytokine producing CD4^+^ T cells **(B)** and CD8^+^ T cells **(C)** are shown. The percentages of responding cells were compared as indicated. Data are representative of two independent experiments with at least four mice per group. *P < 0.05, **P < 0.01, ***P < 0.001, and ****P < 0.0001.

**Figure 5:**
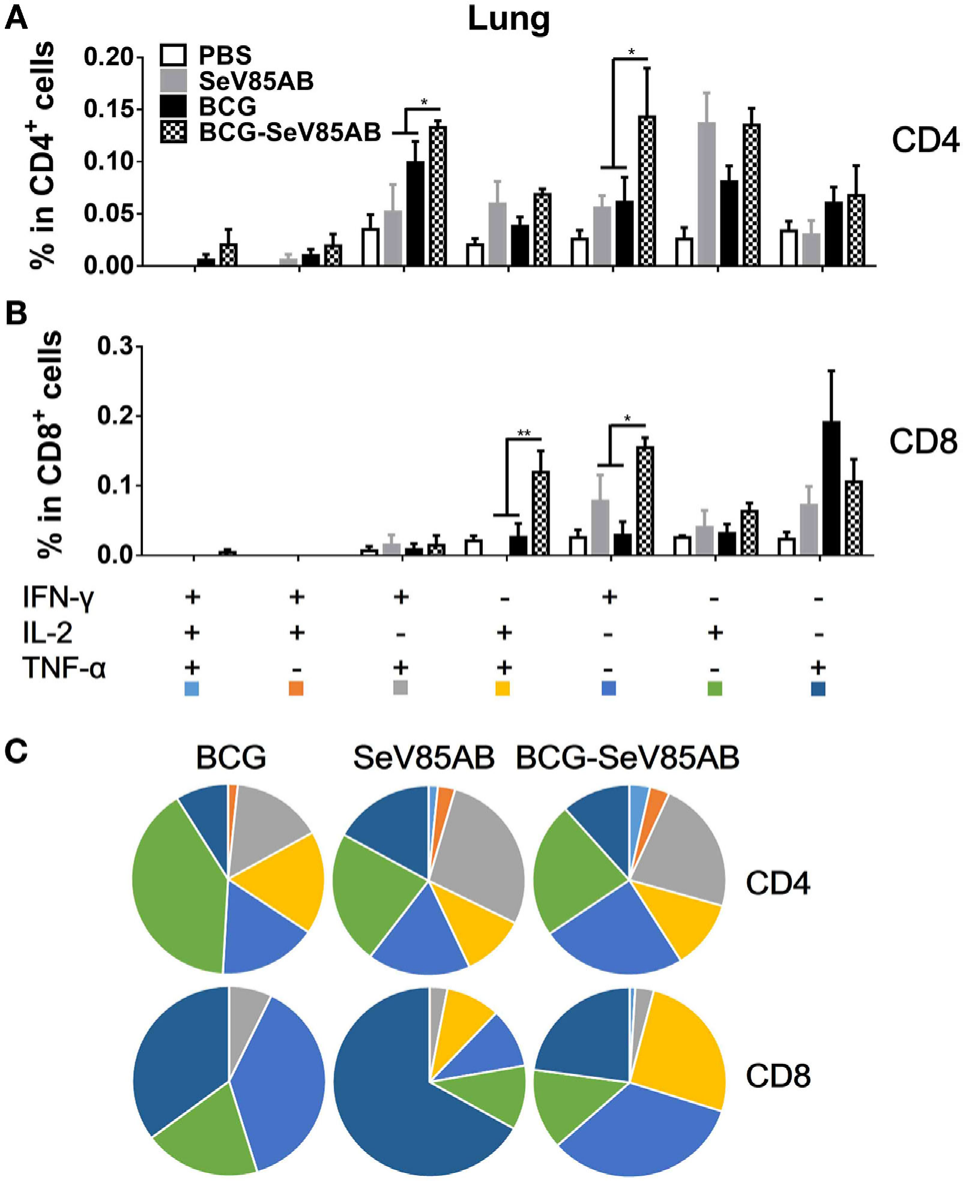
Characterizations of poly-functional Ag85AB-specific T cell responses in the lung. T cells secreting IFN-γ, IL-2, and TNF-α were distinguished as seven distinct subpopulations as described in Figure 3. The percentage of subpopulation as components of the total CD4^+^ **(A)** and CD8^+^ **(B)** T cell populations and the pie chart analysis **(C)** are shown. Significant differences in frequency of poly-functional T cell subsets are indicated. Data are representative of two independent experiments with at least four mice per group. *P < 0.05 and **P < 0.01.

The representative staining results of flow cytometric ICS detection of IFN-γ, IL-2, and TNF-α mono-producing lung T cells after Ag85AB stimulation are shown in Figure 4A and dual-positive plots are shown in Figure S3 in Supplementary Material. The secretion of all three cytokines was significantly boosted, both in CD4^+^ T and CD8^+^ T cells (Figures 4B,C). The poly-functional T cell subset analysis further showed that in CD4^+^ T cells the dual-positive IFN-γ^+^TNF-α^+^ and mono-positive IFN-γ^+^ subsets were significantly boosted (Figures 5A,C), whereas in the CD8^+^ T cells, the IFN-γ^+^ and IL-2^+^TNF-α^+^ subsets were boosted (Figures 5B,C). In the spleen, SeV85AB boost dominantly increased IL-2 responses in CD4^+^ T cells (Figure 3A), whereas in the lung, the greater increases were in IFN-γ positive CD4^+^ T cells (Figure 5B). In the spleen, the CD8^+^ T cell responses had been only modestly enhanced (Figure 3B), whereas in the lung, IL-2^+^TNF-α^+^ and IFN-γ^+^ cells had been significantly increased by SeV85AB (Figure 5B). In addition, PPD-specific CD4^+^ T cell responses in the spleen and CD8^+^ T cell responses in the lung were also boosted by SeV85AB (Figure S4 in Supplementary Material). These divergences confirmed that the mucosal immunization with SeV85AB had a different capacity to establish immune memory in the systemic immune system compared with local site in the lung, confirming our previous observation (15). Taken together, these data demonstrated that SeV85AB boost induced higher levels of poly-functional antigen-specific CD4^+^ and CD8^+^ T cell responses primed by BCG vaccine.

### SeV85AB-Boosted BCG-Induced Immune Protection Was Associated With Recall of Antigen-Specific IL-2-Mediated Responses

**Figure 6:**
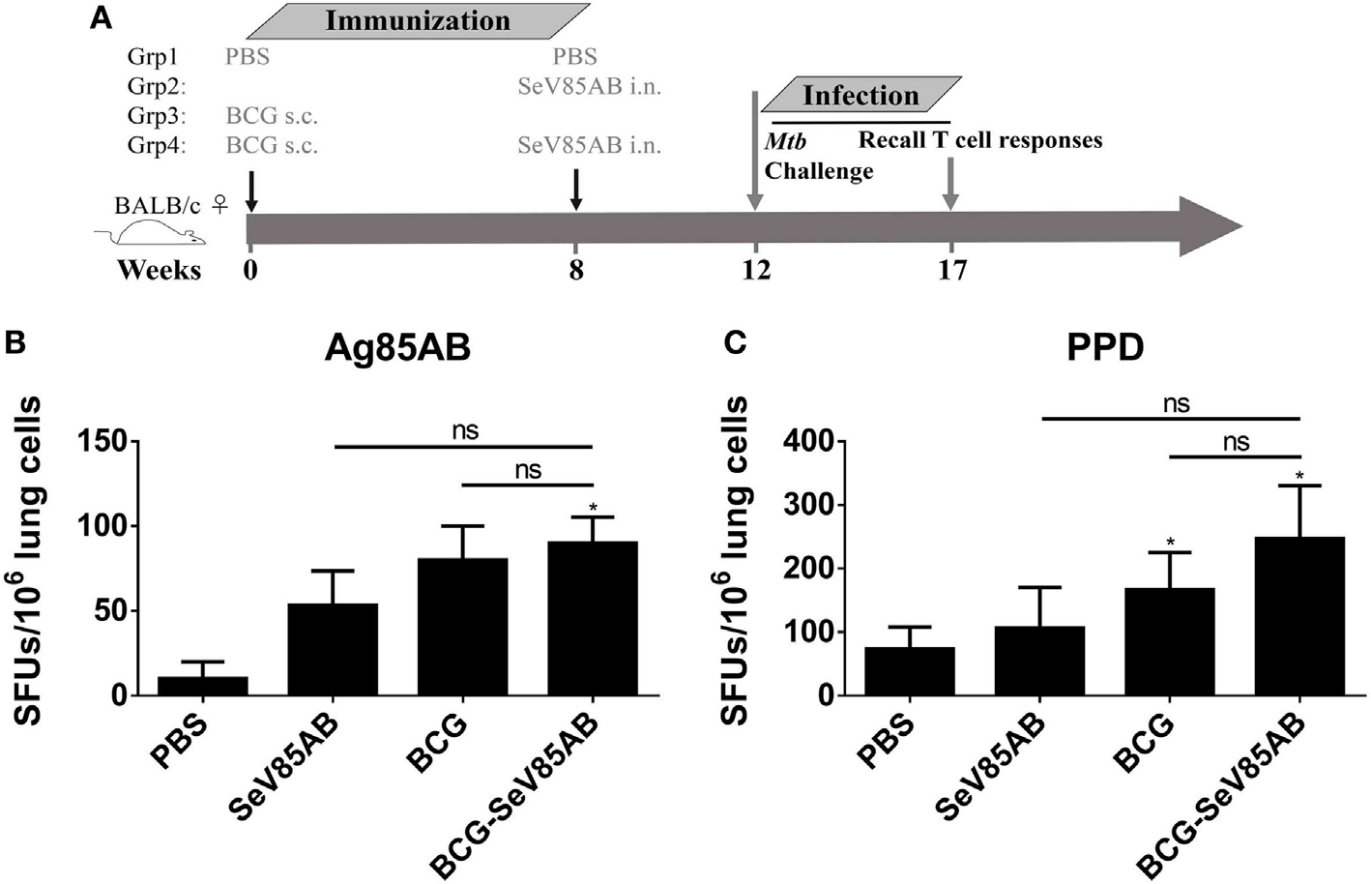
Recall T cell responses against specific stimulation post *Mycobacterium tuberculosis* (*Mtb*) challenge determined by IFN-γ Enzyme Linked Immunospot (ELISPOT) assay. **(A)** Immunization and infection schedule. Vaccinated mice were aerosol challenged 4 weeks later with virulent *Mtb* H37Rv. Five weeks postinfection, recall lung T cell responses were determined. **(B,C)** The IFN-γ responses by ELISPOT assay. Cells from the infected lung were stimulated for 20 h with Ag85AB peptide pool **(B)** or PPD **(C)** in 96-well plates, respectively. Significant differences in frequency between T cell subsets are indicated. Data are representative of two independent experiments with three mice per group. ns, no significant difference.

**Figure 7:**
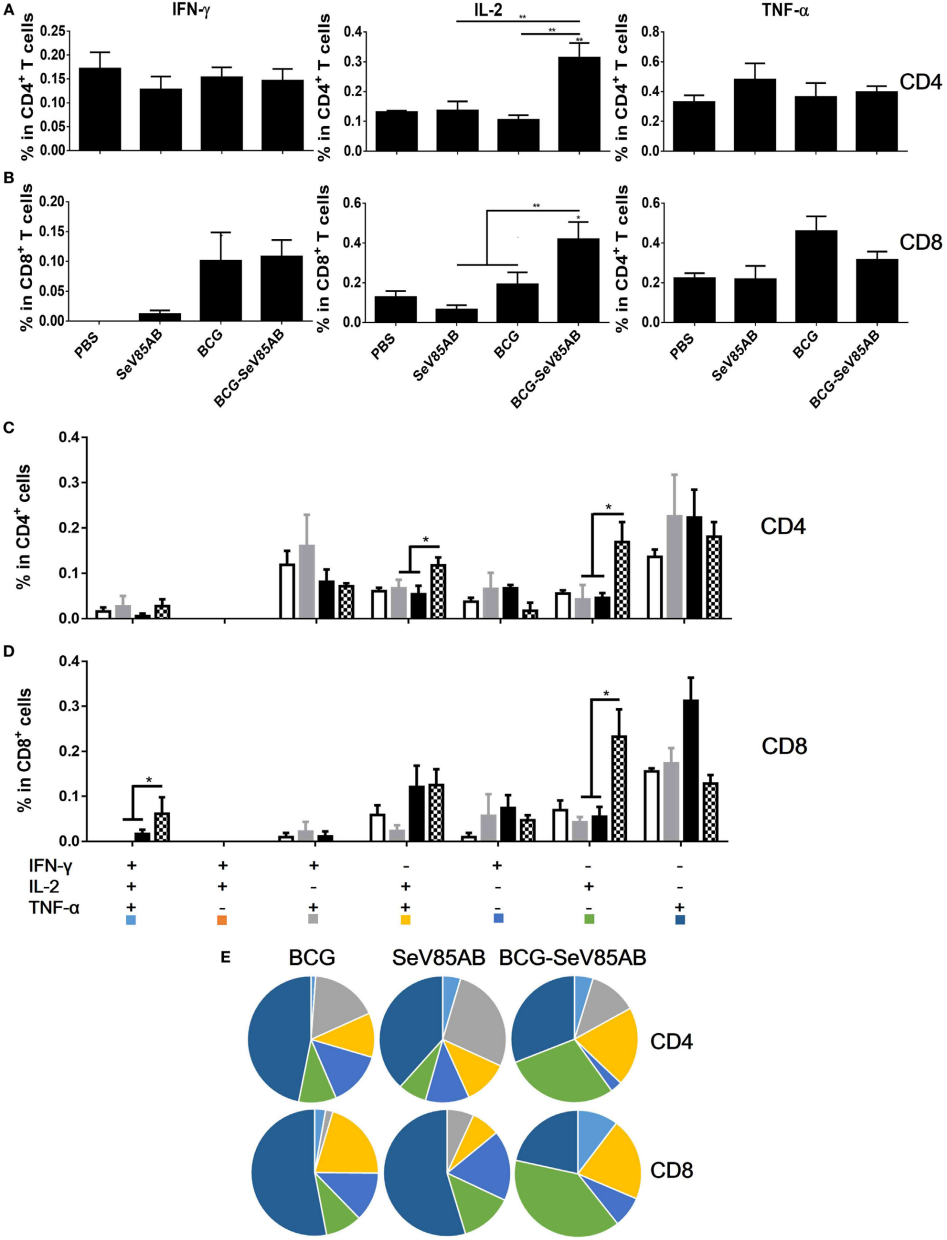
Recall T cell responses against specific stimulation post *Mycobacterium tuberculosis* challenge determined by ICS assay. The immunization and infection schedule were described in Figure 6A. Lung cells were stimulated *ex vivo* with Ag85AB peptides in the presence of monensin and brefeldin A and analyzed for cytokine production by ICS assay. T cells producing IFN-γ, IL-2, and TNF-α were analyzed and their percentage in CD4^+^ T cells **(A)** and CD8^+^ T cells **(B)** are shown. The percentage of seven subpopulations based on the production of three cytokines in any combination in the total CD4^+^ **(C)** and CD8^+^ **(D)** T cells and the pie chart analysis **(E)** are shown. Significant differences in frequency of T cell subsets are indicated. Data are representative of two independent experiments with three mice per group. *P < 0.05 and **P < 0.01.

We had shown that mucosal boosting of SeV85AB improved the protection of BCG vaccination against *Mtb* challenge in a prime-boost model (15). In this study, the protective efficacy afforded by SeV85AB boost was confirmed (data not shown), and the recall T cell responses that occurred upon the *Mtb* infection were especially assayed (Figure 6A). IFN-γ ELISPOT results showed that, at 5 weeks postinfection, mice that had received SeV85AB boosting before the infection had developed only slight, if any, overall increase of Ag85AB- (Figure 6B) and PPD- (Figure 6C) specific responses in the lung compared with the response to infection after SeV85AB or BCG single vaccination. However, SeV85AB boosting resulted in a response to infection that contained a higher percentage of Ag85AB-specific poly-functional lung T cells, notably CD4^+^ T cells with the phenotypes IL-2^+^TNF-α^+^/IL-2^+^, and CD8^+^ T cells of phenotypes IFN-γ^+^IL-2^+^TNF-α^+^/IL-2^+^ (Figure 7).

### SeV85AB Induced Elevated Central Memory Phenotype of CD4^+^ T Cells

**Figure 8:**
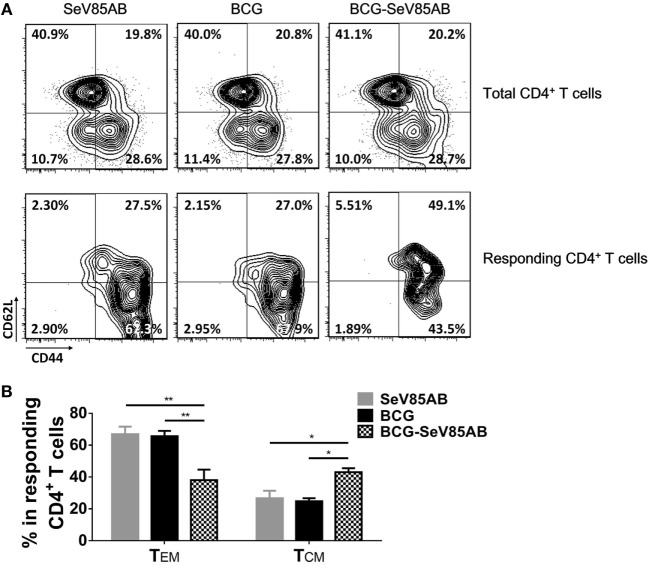
Memory phenotypes of recall T cell responses against *Mycobacterium tuberculosis* challenge. The memory phenotypes in total CD4^+^ T cells and Ag85AB-responding CD4^+^ T cells were assessed. The responding cells were defined as the cells that secreted at least one of the cytokines IFN-γ, IL-2, and TNF-α after Ag85AB peptide stimulation. Central memory T cells (T_CM_) and effector memory T cells (T_EM_) were defined as CD44^+^CD62L^+^ and CD44^+^CD62L^−^, respectively. Representative flow cytometric plots **(A)** and summarized data **(B)** are shown. Data are representative of two independent experiments with three mice per group. *P < 0.05 and **P < 0.01.

The memory phenotypes of antigen-responding CD4^+^ T cells that secreted at least one of the cytokines IFN-γ, IL-2, or TNF-α were further determined (The gating strategy is shown in Figure S5 in Supplementary Material). As a result, SeV85AB boost led to significantly higher levels CD4^+^ T cells with CD44^+^CD62^+^ central memory phenotype compared with other vaccination groups (Figure 8). This was paralleled by decrease in CD44^+^CD62^−^ (effector memory) among those responding CD4^+^ T cells (Figure 8). Thus, our data strongly suggest that the enhanced immune protection induced by SeV85AB boosting was associated with establishment of an increased capacity to recall central memory CD4^+^ T cell responses.

## Discussion

Up to now, most successful vaccines against microbial pathogens have depended on humoral immunity to achieve protection or even sterile eradication. However, as is the case for many intracellular bacteria, *Mtb* is able to resist most antibody-mediated antibacterial effects by growing inside macrophages (23). Thus, T cell-mediated immune responses are crucial for the development of anti-TB vaccines.

The only available anti-TB vaccine remains the almost 100-year-old BCG. The routine administration of BCG to infants provides significant protection against miliary and meningeal TB. However, the protective efficacy is inconsistent in adults. One explanation of BCG’s inadequate protection is a lack of an effective stimulation of an optimal blend of T cell populations (24). Considering that both CD4^+^ and CD8^+^ T cells play important roles in protective immunity against *Mtb* (3, 25, 26), the development of efficient memory CD4^+^ and CD8^+^ T cell responses is one of the main goals of the novel vaccine strategies against *Mtb*.

Although BCG is a strong inducer of Th1 CD4^+^ T cell immune responses, the incidence of active TB disease increases with time after BCG immunization (27), suggesting that a decline of immunological memory after BCG vaccination is one of the causes of the vaccine’s low protective efficacy. However, the waning of BCG protection was not prevented by a BCG revaccination strategy (28). Since the majority of human beings are BCG inoculated, and this seems unlikely to change, a prime-boost regimen is an attractive strategy to counter the waning immune memory post BCG. Heterologous prime-boost vaccination strategy is known to be highly effective for enhancing anti-TB T cell-mediated immunity (17, 29). For example, a vaccinia virus-vectored vaccine (MVA85A) and two adenovirus-based vaccines (AdAg85A and Crucell Ad35) are candidate booster vaccines that are under clinical evaluation. Although MVA85A was shown to be effective in boosting BCG-primed immune protection in a variety of *Mtb* animal infection models, a phase IIb clinical trial indicated that MVA85A may not be effective in humans (30). This failure suggested that novel anti-TB vaccine platforms or optimized delivery systems are urgently needed (31). Improvement would benefit from knowing which cellular responses constitute optimum protective immunity. Our analysis of the cells engaged in the enhanced protective response in the lungs of *Mtb*-challenged mice after BCG/SeV85AB prime/boost is illuminating.

In *Mtb* natural infection, T cells are initially primed in the draining lymph nodes of the infected lung, phagocytosis leads to the presentation and cross-presentation of *Mtb* peptide-loaded MHC-I and -II complexes, which provides the priming “first signal” at the DC surface (32). The “second signal,” a costimulatory signal, tunes the T cell responses by decreasing the activation thresholds of T cells, and pathogen-specific inflammation provides the “third signal” that shapes the maturation of T cells (33–36). A deficiency in any of these signals might lead to the inadequate activation of anti-TB T cell immune memory (37). Sendai virus was chosen here to serve as vector of a booster anti-TB vaccine primarily because it was known that Sendai virus treatment matured dendritic cells and led to complete elimination of tumors cells *in vivo* (38). In addition, Sendai virus tests had demonstrated a potent induction of type I interferon (39) and a Sendai virus-derived RNA agonist of RIG-I had been used as an adjuvant to enhance vaccine-induced immune responses by providing an inflammatory microenvironment (40), thereby optimizing antigen-specific CD4^+^ and CD8^+^ T cell responses (41). Based on this, we initiated this program of investigation and found that the novel Sendai virus vectored vaccine, SeV85AB, did indeed induce robust T cell responses and substantial protection against *Mtb* challenge (15).

Bacille Calmette–Guérin is a strong inducer of systematic CD4^+^ T cell immunity but fails to induce efficient CD8^+^ T cells. In contrast, a single immunization of SeV85AB was able to establish antigen-specific T cells responses and CD8^+^ T cells mediated immune protection (15). Here, we found that the strong antigen-specific CD4^+^ and modest CD8^+^ T cells primed by BCG vaccination were both enhanced by SeV85AB in the spleen. Most notably, the weak antigen-specific CD8^+^ T cell responses that were primed by BCG in the lung were significantly enhanced by the SeV85AB boosting. Heterologous prime-boost and recombinant protein anti-TB vaccine models have shown that the vaccine-induced IL-2-secreting CD4^+^ T cell subsets could maintain the IL-2-producing ability for at least 26 weeks post challenge infection and were associated with enhanced control of bacterial growth in mouse models (42, 43). Increased TNF-α/IFN-γ/IL-2 and decreased TNF-α/IFN-γ responses induced by BCG have been associated with protection against bovine TB (44). In this study, although the IL-2 response was not dominated in the vaccine-induced primary responses in the spleen, IL-2-producing poly-functional T cells were significantly boosted by SeV85AB, supporting the protective role of IL-2 in anti-TB vaccine-induced protection.

Moreover, it was shown that the antigen-specific IL-2^+^CD4^+^ T cell subsets were negative for KLRG1, which is a surface marker of terminally differentiated T cells, during *Mtb* challenge infection (42). We showed previously that the inadequacy of T cell immunity during chronic human TB infection is associated with the less-protective terminally differentiated T cell state marked by KLRG1 expression. Furthermore, the blockade of KLRG1 signaling increased CD4^+^ T cell function through enhancement of Akt pathway in human TB infection (45, 46), supporting the protective role of IL-2 in TB infection. In this study, IL-2-producing T cells were significantly enhanced post *Mtb* infection in the BCG prime-SeV85AB boost regimen group, again showing the recall of IL-2 responses was associated with protective immunity. IL-2 enhances competitiveness for survival in CD4^+^ T cells, thereby facilitating the development of a memory population (47). The CD4^+^ T cells that produce IL-2 could be sustained over a prolonged period and developed into effector cells following antigen stimulation as a result of quick recall response (48). In human TB infection, active TB patients had decreased IL-2-producing CD4^+^ T cells compared with latent TB infection, and 6 months of anti-TB treatment increased specific IL-2-producing T cells (49). In contrast, although also essential for host resistance against *Mtb*, IFN-γ responses do not always correlate with immune protection (26). Recently, Barber et al. also found that IFN-γ accounts for only ~30% of the cumulative CD4^+^ T cell-mediated reduction in lung bacterial loads, but increasing the per capita production of IFN-γ led to the early death of the host in murine TB infection (20). Thus, although it can be regarded as a positive marker of vaccine-induced primary T cell responses, the degree of infection-induced IFN-γ production might not always be the factor limiting immune protection against TB. Instead, our data support a key positive role of IL-2 in anti-TB immune protection. Thus, perhaps recall IL-2-mediated, instead of IFN-γ-mediated T cell responses is the critical factor normally limiting protection against TB infection.

Cumulatively, our data suggest that boosting BCG with SeV85AB might compensate for the weak induction by BCG of IL-2-dependent recall T cell immune responses in the lung. Since we showed here that an SeV85AB boost significantly enhanced the T cell immune memory induced by BCG vaccination in mice, further studies of SeV85AB in other animal models are warranted before a clinical trial of safety in humans.

## Ethics Statement

This study was approved by the Institutional Animal Care and Use Committee and was performed according to the guidelines of the Laboratory Animal Ethical Board of Shanghai Public Health Clinical Center.

## Author Contributions

X-YF and TS conceive the project. X-YF and ZH designed the study, analyzed the data, and wrote the manuscript. ZH, LG, and C-LL performed the experiments. DL provided editorial input and improved the writing.

## Conflict of Interest Statement

X-YF, TS, ZH, and DL are co-inventors of a patent application on the novel SeV85AB vaccine.
