# Supplementary material for: The Profile of T Cell Responses in Bacille Calmette–Guérin-Primed Mice Boosted by a Novel Sendai Virus Vectored Anti-Tuberculosis Vaccine
Source: Front Immunol. 2018 Aug 3;9:1796. doi: 10.3389/fimmu.2018.01796 (PMC6085409; doi:10.3389/fimmu.2018.01796)
Supplement: Supplementary file 1 [file Table_1.docx]

**The Profile of T Cell Responses in BCG-Primed Mice Boosted by a Novel Sendai Virus Vectored Anti-Tuberculosis Vaccine**

**Zhidong Hu^1^, Ling Gu^1^, Chun-Ling Li^2^, Tsugumine Shu^3^, Douglas B. Lowrie^1,2^, Xiao-Yong Fan^1,2*^**

*^1^ Shanghai Public Health Clinical Center, Key Laboratory of Medical Molecular Virology of MOE/MOH, Fudan University, 2901 Caolang Rd., Shanghai 201508, China;*

*^2^ School of Laboratory Medicine and Life Science, Wenzhou Medical University, Wenzhou 325035, China;*

*^3^ ID Pharma, Ibaraki 300-2611, Japan.*

^*^ Correspondence: Dr. Xiao-Yong Fan ([xyfan008@fudan.edu.cn](mailto:xyfan008@fudan.edu.cn)).

**Conflict of interest:** X.Y.F., T.S., Z. H., and D.B.L. are co-inventors of a patent application on the novel SeV85AB vaccine.

# Supplementary Figures

**
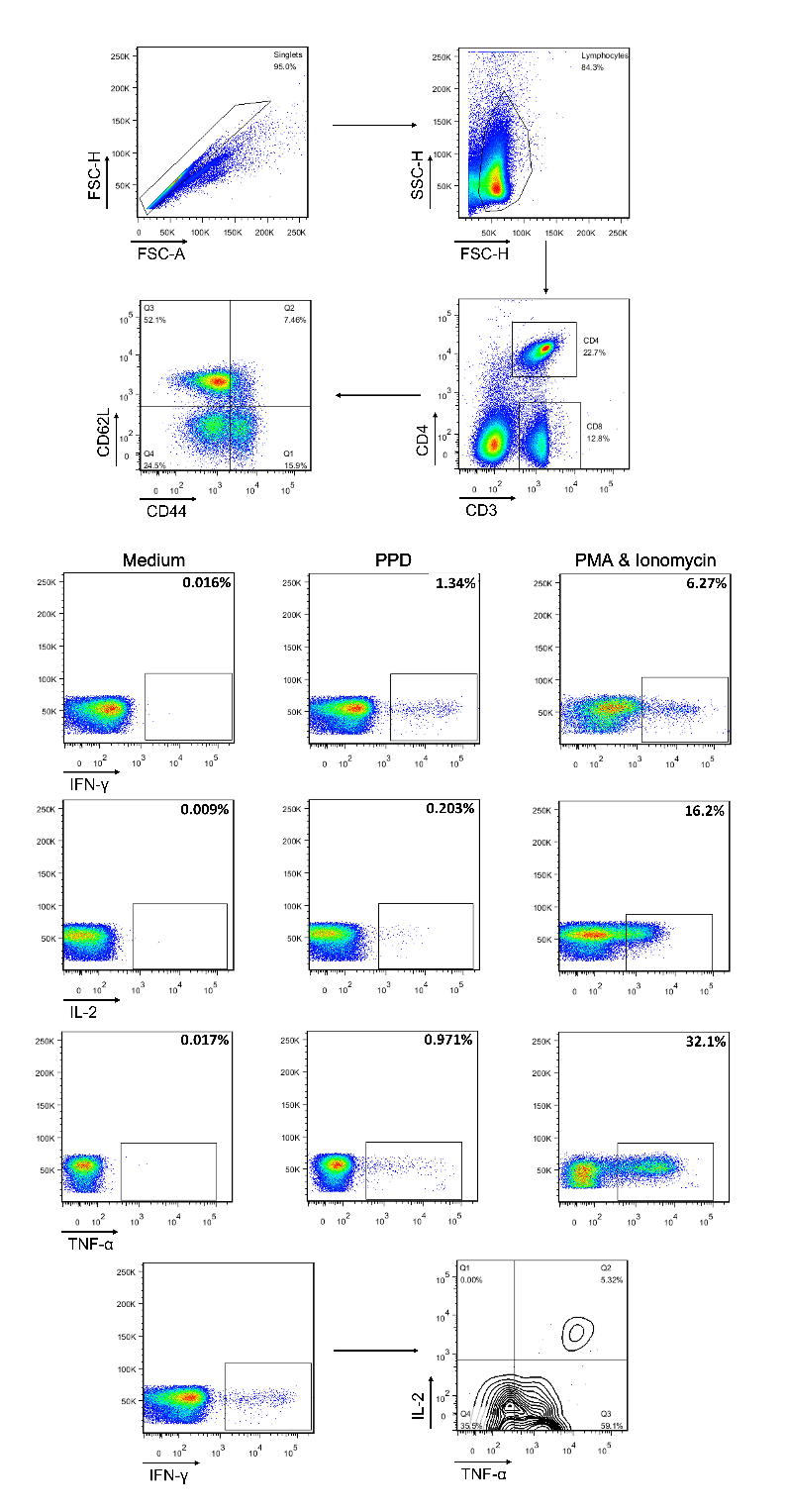
**

**Figure S1.** Gating strategy in ICS assay. Representative flow cytometric plots are shown. Briefly, CD4^+^ T cells were gated as CD3^+^CD4^+^ cells and CD8^+^ T cells were defined as CD3^+^CD4^-^ cells. The cells were stimulated with PPD or Ag85AB peptide pools, with medium as negative control, and PMA (50 ng/ml, Sigma) plus ionomycin (1 µg/ml, Sigma) stimulation as positive control. The expression of intracellular cytokines and T cell memory phenotypes were detected with appropriate antibodies.





**Figure S2.** Typical dual-positive dot plots in ICS assays using splenocytes. After Ag85AB peptides or PPD stimulation, the expression of IFN-γ, IL-2 and TNF-α was detected. The representative dual-positive dot plots in CD4^+^ T cells are shown.





**Figure S3.** Typical dual-positive dot plots in ICS assays using lung cells. After Ag85AB peptides or PPD stimulation, the expression of IFN-γ, IL-2 and TNF-α were detected. The representative dual-positive dot plots in CD4^+^ T cells are shown.





**Figure S4.** Characterizations of poly-functional PPD-specific T cell responses. Four weeks after vaccination, cells from the spleen (A, C) and lung (B, D) were stimulated for 5 h with PPD (10 μg/ml) in presence of Monensin and Brefeldin A. Frequency of poly-functional cytokines-producing CD4^+^ (A, B) and CD8^+^ (C, D) T cells were determined by ICS assay. The frequencies of poly-functional T cells were compared as indicated. * *P* < 0.05.





**Figure S5.** Gating strategy of memory phenotype determination in total or responding CD4^+^ T cells. Representative flow cytometric plots are shown. Briefly, CD4^+^ T cells were gated as CD3^+^CD4^+^ cells. The memory phenotypes were determined in total CD4^+^ T cells and in antigen-responding CD4^+^ T cells. The latter were defined as CD4^+^ T cells that secreted at least one of the cytokines IFN-γ, IL-2 or TNF-α post Ag85AB peptides stimulation. CD44^+^CD62^+^ and CD44^+^CD62^-^ cells defined central memory and effector memory T cells, respectively.
